# Impact of a multifaceted intervention programme on antibiotic prescribing in out-of-hours primary care in five European countries: secondary analyses of the HAPPY PATIENT project

**DOI:** 10.1093/jacamr/dlag219

**Published:** 2026-10-16

**Authors:** Ingrid Keilegavlen Rebnord, Carl Llor, Fabiana Raynal Floriano, Malene Plejdrup Hansen, Jesper Lykkegaard, Jonas Olsen, Pia Touboul-Lundgren, Pascale Bruno, Anna Kowalczyk, Christos Lionis, Ruta Radzeviciene, Lina Jaruseviciene, Lars Bjerrum, Ana Garcia-Sangenis, Bent Håkan Lindberg

**Affiliations:** Department of Global Public Health and Primary Care, University of Bergen, Bergen, Norway; NORCE Norwegian Research Centre AS, Bergen, Norway; University Institute in Primary Care Research Jordi Gol, Barcelona, Spain; CIBER de Enfermedades Infecciosas, Instituto de Salud Carlos III, Madrid, Spain; Department of Public Health, Research Unit for General Practice, University of Southern Denmark, Odense, Denmark; Quantitative Methods for Economics and Management, University of Las Palmas de Gran Canaria, Las Palmas de Gran Canaria, Spain; Department of Public Health, Research Unit for General Practice, University of Southern Denmark, Odense, Denmark; Center for General Practice at Aalborg University, Aalborg, Denmark; Department of Public Health, Research Unit for General Practice, University of Southern Denmark, Odense, Denmark; Department of Public Health, Research Unit for General Practice, University of Southern Denmark, Odense, Denmark; Department of Public Health, Nice University Hospital, Nice, France; Department of Public Health, Nice University Hospital, Nice, France; Centre for Family and Community Medicine, Faculty of Health Sciences, Medical University of Lodz, Lodz, Poland; Clinic of Social and Family Medicine, School of Medicine, University of Crete, Heraklion, Greece; Department of Psychology, School of Social Sciences and Humanities, University of Limassol, Limassol, Cyprus; Ltd Mano Seimos Gydytojas (My Family Doctor), Klapeida, Lithuania; Department of Family Medicine, Lithuanian University of Health Sciences, Kaunas, Lithuania; Section of General Practice, Department of Public Health, University of Copenhagen, Copenhagen, Denmark; University Institute in Primary Care Research Jordi Gol, Barcelona, Spain; NORCE Norwegian Research Centre AS, Bergen, Norway; Department of General Practice, Institute of Health and Society, University of Oslo, Oslo, Norway

## Abstract

**Background:**

Antimicrobial resistance is a major global health threat, driven mainly by excessive and inappropriate antibiotic use. As most antibiotics are prescribed outside hospitals, primary care measures are crucial for reducing use. The study aimed to investigate diagnostic coding and antibiotic prescribing for respiratory tract (RTI) and urinary tract (UTI) infections treated in out-of-hours clinics before and after a multifaceted intervention.

**Methods:**

Audit and feedback with analyses of prescription data from five out-of-hours clinics in Europe before and after a multifaceted intervention. The intervention consisted of an audit with reports and feedback to the prescriber, participation in workshops, communication and e-learning courses, and distribution of information materials to patients.

**Results:**

A total of 67 physicians recorded 1786 cases in the first period and 1820 in the second. Common cold/influenza was the most frequent diagnosis, increasing from 19.4% (95% CI 18–21) in the first to 28.7% (95% CI 27–31) in the second period. COVID-19 declined by 65.7% (*P* < 0.001), while pharyngotonsillitis increased by 31.7% (*P* = 0.001). The intervention did not alter the overall antibiotic prescribing rate for RTIs (49.3% versus 48.7%, *P* = 0.748), and more than three-quarters of RTI prescriptions were judged unnecessary or inappropriate. For UTIs, the use of first-line antibiotics increased significantly from 70.0% to 79.5% (*P* = 0.027).

**Conclusions:**

The study provided important insights into which RTIs are potentially inappropriately treated with antibiotics, although the intervention did not produce a statistically significant reduction in antibiotic prescribing. Positively, the use of first-line antibiotics for UTIs increased significantly.

## Introduction

Antibiotic resistance is a significant threat to public health worldwide, and antibiotic use is one of the main drivers.^1^ A significant proportion of total human antibiotic consumption is prescribed in primary healthcare; however, there is considerable variation between countries.^2^ Physician-, patient- and healthcare system–related factors influence prescribing decisions, and several antibiotic stewardship programmes have been introduced to reduce inappropriate antibiotic use.^3^ Hence, interventions to reduce inappropriate antibiotic prescribing, targeting healthcare personnel and the public, are pertinent, necessary and wanted.^4,5^

Out-of-hours (OOH) primary care is essential for a well-functioning healthcare system, as people’s perceived need for health assessment can arise at any time. The European Union (EU) and other Organisation for Economic Co-operation and Development countries organize their OOH services in different ways, and there are several models within countries.^6,7^ General practitioners’ (GPs) cooperatives are the most common model in the EU.^7^ Most countries’ OOH clinics serve all age groups, while some countries offer dedicated paediatric services.

Despite organizational differences, there is a high consistency in the OOH epidemiology across European countries, with high attendance for acute respiratory tract infections (RTIs).^8^ These highly prevalent infections are typically self-limiting and seldom benefit from antibiotic treatment.^9^ European national guidelines recommend restricted prescribing practices, and RTIs are essential targets for interventions to reduce inappropriate antibiotic prescribing.^10^

Symptoms of urinary tract infections (UTIs) are unpleasant and tend to appear abruptly, which may explain why these infections are frequently managed in OOH primary care. Antibiotics are indicated for UTIs; however, prescribers should select narrow-spectrum antibiotics, taking local antimicrobial resistance patterns into account.^10^

The OOH setting differs from the daytime general practice setting in that patients are unknown, consultations occur at irregular hours and referral options are usually fewer. These factors influence how physicians perceive their work and prescribe antibiotics.^11,12^ Whether RTI OOH populations across different healthcare systems have higher acuity and more antibiotic-demanding infections than regular-hours patients remains unresolved.^11,13,14^

Multifaceted interventions have been more effective than single interventions in reducing unnecessary antibiotic prescribing.^15,16^ Audit Project Odense (APO) is an internationally accepted approach for quality improvement, built on audit and feedback for participating healthcare personnel.^17,18^ Interventions using the APO method have led to marked reductions in antibiotic prescribing within different countries across several continents.^19–21^

The project Health Alliance for Prudent Prescribing and Yield of Antibiotics in a patient-centred Perspective (HAPPY PATIENT) was a prospective, non-randomized, multifaceted intervention conducted in five countries: France, Greece, Lithuania, Poland and Spain. It investigated, for the first time, the effect of the APO method on antibiotic prescribing for RTIs and UTIs in OOH primary care and found potentially unnecessary antibiotic prescribing in 52% of the contacts.^21–23^

This study aims to provide an in-depth analysis of how an intervention built on the APO methodology influenced diagnostic coding and antibiotic treatment of common infections in the OOH setting in five European countries.

## Methods

### Study design

The participating countries organize their OOH services in various ways. The main differences were whether the services were organized within primary or secondary care, the physicians’ specialities and the availability of point-of-care tests. Despite these differences, all included clinics were the first point of contact for patients who perceived a need for OOH medical assessment. A pilot test of the registration material was conducted in November 2021 to confirm that the registration chart was relevant to their practice and readily comprehensible. According to the study protocol, we aimed to recruit at least 25 OOH physicians per country. The calculation assumed a baseline antibiotic prescribing rate of 40%, a reduction to 25%, an intracluster correlation coefficient of 0.1, *α* = 0.05 and 80% power, requiring 25 healthcare professionals per audit cycle.^23^ The present analysis included all eligible cases recorded in the five participating OOH clinics during the two study periods: the pre-intervention registration from February to April 2022 and the post-intervention registration from February to April 2023 with no separate *post hoc* sample size calculation.

### Registrations and variables

The participants used APO paper-based registration forms with predefined categories (Figure S1, available as Supplementary data at *JAC-AMR* Online). For each patient, we recorded patient age, sex, duration of symptoms, predefined categories of symptoms and clinical findings, use of point-of-care tests, diagnosis, whether antibiotic treatment was initiated and, if so, which type and whether admitted to the hospital.

The primary outcome was whether antibiotics were prescribed. We then dichotomized antibiotics into first- and second-line, according to each country's national guidelines (Figure S2). Second-line antibiotics were defined as inappropriate for all infections.

Unnecessary antibiotic prescriptions were defined using an algorithm developed for this study (Figure S2). For common cold/influenza, COVID-19 and acute bronchitis/bronchiolitis, all prescriptions were deemed unnecessary. For rhinosinusitis, a symptom duration of <7 days deemed a prescription unnecessary. For acute otitis media, symptom duration and patient age were decisive, while sore throat and at least two CENTOR criteria defined the basis for assessing diagnoses and treatment for acute pharyngotonsillitis. If multiple diagnoses were present, the prescription was deemed necessary if one of the diagnosis met the criteria.

Only physicians who participated in both registration periods were included in this study.

### Intervention

The pragmatic intervention took place from October 2022 to February 2023 and had the following elements:

1. An individual report of the results from the first registration with feedback on the prescribing of the individual and the national group, facilitating reflections on their results with peer-to-peer feedback and group discussions about any quality issues.
2. Participation in a workshop to discuss strategies for quality development and knowledge exchange on antibiotic prescribing.
3. A 2-h training session on communication skills, held online in France, while the other countries had both physical and online meetings.
4. Distribution of written patient information materials, prepared and adjusted to the local setting by the HAPPY PATIENT team, based on the project’s Delphi study.^24^ It included patient brochures and handouts to identify and address misconceptions and knowledge gaps about antibiotic use.
5. Access to an e-learning platform with clinically relevant cases for the out-of-hours setting, focusing on appropriate use of antibiotics for common infections, updated guidelines and evidence-based recommendations for diagnosis and treatment.

All participants were provided with the complete set of information materials and feedback. They were encouraged to participate in workshops and training sessions, but those not participating were also included. How these meetings were offered per country is described in Supplementary data (Table S1).

### Statistical analyses

We summarized descriptive statistics using averages for continuous variables and percentages for categorical variables.

To compare the percentages of different variables between physicians, we used χ^2^ tests for categorical variables and Student’s *t*-tests for continuous variables. We assessed comparability between the two registration periods by comparing patient characteristics and prescription rates across the two registration rounds to determine whether significant differences existed between the samples. We analysed prescribing using generalized estimating equations (GEEs) with a binomial distribution, a logit link function, and an independent working correlation structure. Physician (participant number) was specified as the clustering variable to account for correlation among patients treated by the same physician. The model was adjusted for patient age and the eight RTI diagnoses with common cold/influenza as the reference category. Effect estimates are reported as odds ratios (OR) with 95% Wald CIs. We then ran separate GEE models to estimate diagnosis-stratified adjusted OR for prescribing before and after the intervention. The significance level was set at *P* < 0.05.

IBM SPSS Statistics (Version 29.0.2.0 (20)) was used for the statistical analyses.

### Ethics

The ethics committee boards of the five participating countries assessed the study. The boards of Greece and Spain approved the project, while the boards in Lithuania, Poland and France deemed it did not require approval. Participants signed consent after being informed about the study and that they could withdraw at any time. All information about patients was non-identifiable.

## Results

A total of 67 physicians participated in both registration periods (19 physicians dropped out after the first registration). They registered 1786 cases in the first period and 1820 cases in the second. The patients’ median age was 36 years [interquartile range (IQR) 20–60] in the first registration period and 32 years (IQR 14–55) in the second (Table 1). The most frequent diagnosis was common cold/influenza in both periods. The diagnostic distribution shifted between the two periods, with a 65.7% reduction in COVID-19 cases (*P* < 0.001) and a 31.7% increase in pharyngotonsillitis cases (*P* = 0.001). The admission rate was statistically significantly lower in the second period, with a 30.1% decrease (*P* < 0.001) and was significant for pneumonia (from 71.9% to 56.4%; *P* = 0.003) and COVID-19 (from 40.7% to 18.0%; *P* < 0.001).

**Table 1.** Patient characteristics, diagnoses, antibiotics prescribed and hospital admittance in the two registration periods

Variables	Before		After		*P* value
	(*n* = 1786)		(*n* = 1820)		
	*n*	%	*n*	%	
Sex^a^					
Female	1000	56	1044	57.4	0.385
Male	786	44	774	42.6	
Age groups^a^					
0–17 years	383	21.7	506	27.9	<0.001
18–64 years	1003	56.8	993	54.8	
>65 years	381	21.6	313	17.3	
Duration of symptoms^b^	Mean 3.71 Median 3.0	SD 4.14	Mean 3.36 Median 3.0	SD 3.17	0.005
Symptom and signs^a^					
Fever	889	49.8	995	54.7	0.003
Sore throat	664	37.2	726	39.9	0.094
Cough	877	49.1	895	49.2	0.966
Purulent sputum increased	183	10.2	183	10.1	0.849
Ear pain	111	6.2	126	6.9	0.391
Tonsillar exudate	149	8.3	217	11.9	<0.001
Tender cervical adenopathy	109	6.1	181	9.9	<0.001
Tachypnoea	176	9.9	140	7.7	0.022
Abnormal lung auscultation	350	19.6	308	16.9	0.038
Dysuria	285	16.0	293	16.1	0.908
Flank pain	172	9.6	127	7.0	0.004
Poor general condition	316	17.7	264	14.5	0.009
Tests performed^a^					
Strep-A test	98	5.5	165	9.1	<0.001
C-reactive protein test	695	38.9	720	39.6	0.691
COVID-19 test	861	48.2	483	26.5	<0.001
Urinary dipstick	316	17.7	346	19.0	0.307
Diagnoses^a^					
Common cold/influenza	347	19.4	522	28.7	<0.001
COVID-19	317	17.8	111	6.1	<0.001
Acute pharyngotonsillitis	226	12.6	302	16.6	0.001
Pneumonia	185	10.4	163	9.0	0.154
Acute bronchitis/bronchiolitis	149	8.3	175	9.6	0.181
Acute otitis media	69	3.9	89	4.9	0.132
Acute rhinosinusitis	59	3.3	57	3.1	0.770
Exacerbation of COPD	39	2.2	34	1.9	0.501
Urinary tract infection	237	13.3	213	11.7	0.155
Pyelonephritis	120	6.7	99	5.4	0.108
None of the above	146	8.2	118	6.5	0.051
Antibiotics prescribed^a^	880	49.3	887	48.7	0.748
First-line antibiotics	359	20.1	371	20.4	0.832
Second-line antibiotics	386	21.6	365	20.1	0.249
NA^c^	135		151		
Admittance to hospital^a^	332	18.6	236	13.0	<0.001

^a^χ^2^  *P* value.

^b^Student’s *t*-test, *P* value.

^c^Not available (NA) for categorizing, either ticked of ‘other antibiotics’ or double prescription.

Overall antibiotic prescribing rates declined by 1.2% (*P* = 0.748). The use of first-line antibiotics increased by 1.5% (*P* = 0.832), while second-line antibiotic prescribing decreased by 6.9% (*P* = 0.249). Symptom duration prior to clinical contact was 3.4 in the first period compared with 3.7 days in the second, a significant but not clinically relevant reduction of 0.3 days (*P* = 0.005).

### Respiratory tract infections

A total of 2499 patients were diagnosed with an RTI. The proportion of patients prescribed antibiotics did not differ significantly before and after the intervention (OR = 1.06; 95% CI 0.74–1.55; *P* = 0.764; Table 2). Increasing age was associated with a higher probability of a prescription (OR = 1.01 per year; 95% CI 1.00–1.02; *P* = 0.004). Diagnosis strongly predicted prescribing: compared with common cold/influenza (reference), patients with all other diagnoses had significantly higher odds of being prescribed antibiotics. Because a period × diagnosis interaction term added to the adjusted model was statistically significant (Wald χ2 = 267.4, df = 14, *P* < 0.001), indicating that the effect of the intervention on prescribing differed by diagnosis, separate GEE models (period and age, physician as clustering variable) were subsequently fitted within each diagnosis category. Prescribing decreased statistically significantly after the intervention for COVID-19 (OR = 6.80, 95% CI 2.35–19.65; *P* < 0.001), whereas it increased for pneumonia (OR = 0.21; 95% CI 0.10–0.46; *P* < 0.001). No before–after difference was found for the remaining diagnoses. The total number of unnecessary and inappropriate prescriptions was not statistically significantly reduced for any RTI diagnosis following the intervention (Table 3). In contrast, it increased between the two periods for acute pharyngotonsillitis and exacerbation of COPD, and 88% of the RTI prescriptions were possibly unnecessary or inappropriate.

**Table 2.** Adjusted odds ratios for antibiotic prescribing (GEE, binomial/logit and physician as clustering variable)

Variable	OR^a^	95% CI	*P* value
Period: before intervention (ref. = after)	1.06	0.74–1.55	0.764
Age (per year)	1.01	1.00–1.02	0.004
Diagnosis			
COVID-19	5.95	1.84–19.22	0.003
Acute otitis media	108.20	40.61–288.59	<0.001
Acute rhinosinusitis	52.30	22.49–121.63	<0.001
Acute pharyngotonsillitis	130.19	51.32–329.97	<0.001
Acute bronchitis/bronchiolitis	28.50	11.46–70.81	<0.001
Pneumonia	272.87	103.03–723.43	<0.001
Exacerbation of COPD	82.85	30.02–228.61	<0.001
Common cold/influenza (reference)	1.00	—	—
Diagnosis-stratified odds ratios for prescribing before versus after intervention^b^			
COVID-19	6.80	2.35–19.65	<0.001
Common cold/influenza	0.56	0.25–1.27	0.167
Acute otitis media	1.47	0.62–3.49	0.389
Acute rhinosinusitis	0.68	0.27–1.73	0.419
Acute pharyngotonsillitis	0.76	0.45–1.30	0.318
Acute bronchitis/bronchiolitis	1.57	0.86–2.86	0.140
Pneumonia	0.21	0.10–0.46	<0.001
Exacerbation of COPD	0.97	0.31–3.06	0.958

OR, odds ratio; CI, confidence interval.

^a^The reference category is ‘not prescribed’. The model is adjusted simultaneously for period, age and diagnosis.

^b^OR for the period before relative to after (ref. = after). OR > 1 indicates higher prescribing before the intervention. OR < 1 indicates higher prescribing after.

**Table 3.** Unnecessary and/or inappropriate antibiotic prescription

Diagnoses	Prescription before number			Unnecessary and inappropriate %	Prescription after number			Unnecessary and inappropriate %	*P* value
	Unnecessary	Inappropriate decision	Total prescription		Unnecessary	Inappropriate decision	Total prescription		
Common cold/influenza	6	1	7	100.0	18	1	19	100.0	NA
COVID-19^a^	56	32	90	93.6	5	1	6	100.0	0.712
Acute pharyngotonsillitis	53	73	156	80.8	84	117	226	88.9	0.025
Pneumonia	57	77	147	91.2	81	54	153	88.2	0.406
Acute bronchitis/bronchiolitis^a^	65	3	68	100.0	57	4	63	96.8	0.139
Acute otitis media	41	7	54	88.9	38	11	62	79.0	0.152
Acute rhinosinusitis	28	4	33	97.0	22	10	36	88.9	0.196
Exacerbation of COPD	7	7	31	45.2	11	12	27	85.2	0.002
Sum	313	204	586	88.2	316	210	592	88.9	0.437

^a^Multiple diagnoses present, the prescription was deemed necessary if one of the dignoses fulfilled the criteria.

In the group diagnosed with acute pharyngotonsillitis, the sum of CENTOR criteria increased slightly among patients who received a prescription, from 2.58 to 2.82 (*P* = 0.024), but unnecessary/inappropriate prescriptions still increased. In the first period, 23.5% of patients had 0 or 1 CENTOR criteria fulfilled, and in the second period, 19.2%, indicating a high number of probably unnecessary prescriptions in addition to the high share of second-line antibiotics prescribed for this diagnosis. Fever, lack of cough and tonsillar exudate were the most frequent criteria but only 11.9% had all four criteria fulfilled in the first period and 19.2% in the second period (Table S4).

For acute otitis media and rhinosinusitis, short symptom duration and few cases with fever or poor general condition were the main reasons prescriptions were deemed unnecessary (Tables S7, S8).

Prescriptions for pneumonia increased statistically significantly, while inappropriate prescriptions showed a non-significant decrease. On the other hand, inappropriate prescriptions increased significantly for COPD exacerbations.

### Urinary tract infections

A total of 592 patients were diagnosed with either cystitis or pyelonephritis; 11 patients were diagnosed with both UTI diagnoses and 66 with both RTI and UTI diagnoses. Table 4 shows the distribution of gender, symptoms and clinical findings and the use of relevant tests for those with only one UTI diagnosis. Significantly more patients underwent urinary dipstick testing after the intervention, from 66.2% to 80.0% (*P* = 0.002), and first-line antibiotic use in the cystitis group increased significantly from 70.0% to 79.5% (*P* = 0.027).

**Table 4.** Patients diagnosed with only one UTI diagnosis

	Before		After		*P* value
	*n* = 302		*n* = 290		
Age^a^	Mean 43.44 Median 38.00	SD 23.65	Mean 44.86 Median 40.00	SD 23.83	0.470
	*n*	%	*n*	%	
Sex^b^					
Female	239	79.1	233	80.3	0.715
Symptom^b^					
Dysuria	234	77.5	248	85.5	0.012
Flank pain	87	28.8	67	23.1	0.114
Fever	91	30.1	87	30.0	0.972
Poor general condition	52	13.9	54	18.6	0.120
Test^b^					
Urinary dip stick	209	69.2	239	82.4	<0.001
C-reactive protein test	104	34.4	127	43.8	0.020
Antibiotic^b^					
Cystitis	*n* = 210		*n* = 200		
First-line antibiotics	147	70.0	159	79.5	0.027
Second-line antibiotics	63	30.0	41	20.5	
Pyelonephritis	*n* = 92		*n* = 90		
First-line antibiotics	2	2.2	7	7.7	0.081
Second-line antibiotics	90	97.8	83	92.2	
Admitted to hospital	40	13.2	52	17.9	0.116

^a^Student’s *t*-test, *P* value.

^b^χ^2^  *P* value.

## Discussion

### Main findings

After adjustment for age, diagnostic case-mix and physician-level clustering, we found no evidence of an overall change in antibiotic prescribing following the intervention. However, first-line antibiotic use for cystitis increased in the second period. The study provided valuable information on patients’ reported symptoms when visiting participating physicians in European out-of-hours clinics, the diagnostic tools used and the factors that influenced decisions to prescribe antibiotics.

### Strengths and limitations

This study employed a pre–post design without a control group, limiting causal inference. Recruitment in the OOH setting was challenging, and the target of 25 physicians per country, each registering 25 cases across two periods (totalling 3125 infections), was not achieved. Participation was particularly low in France and Greece, with only Lithuania meeting the recruitment goal.

These challenges reflect the heterogeneous organization of OOH services across countries, ranging from hospital-based to private or public primary care clinics.^7^ Overall prescription rates are difficult to compare across countries due to variations in prescribing culture and the organization of OOH services. Many physicians work OOH shifts in addition to other roles, often for short periods, complicating consistent participation. Because the APO methodology requires the same physicians to register cases before and after the intervention, we included only those who participated in both registration periods in the analyses.

A central feature of the APO methodology is the standardized registration template, in which participants tick predefined variables. The limited number of variables and the simplicity of the form, which allows only binary (yes/no) responses, facilitate ease of use during busy clinical hours and ensure consistent data collection, both of which are strengths. However, this simplicity also restricts the depth of information, particularly regarding cultural or non-biomedical factors such as perceived pressure to prescribe antibiotics. The small number of variables also posed challenges in capturing both RTIs and UTIs, which differ in symptoms and clinical findings. Laboratory test variables were especially limited, as only the test performance could be recorded, not the result, thereby reducing their analytical value.

Participation was voluntary, which may introduce selection bias. Those who chose to participate may have had a greater interest in antibiotic stewardship and quality improvement and may have been more inclined to change prescribing habits.^25^ While self-registration could potentially influence prescribing behaviour, previous studies have shown that the APO methodology correlates well with actual prescribing practices.^18^ Self-registration carries risks of error and selective reporting. Treatment choices may also influence diagnostic decisions, although this bias likely affected both periods equally, thereby minimizing its effect on the measured impact of the intervention.^26^

The study’s multinational scope, spanning five countries with diverse healthcare systems, antibiotic use patterns and patient expectations, enhances generalizability. However, external factors such as the COVID-19 pandemic, a rise in invasive group A streptococcal infections and an increase in influenza epidemics in 2023 may have influenced both participation and prescribing behaviour. Additionally, shortages of first-line antibiotics in Lithuania and Poland may have affected the expected reduction in second-line antibiotic use.

### Discussion of results/implications for practice

The study population differed notably between the two periods. In the latter period, a higher proportion of children aged 0–17 years was observed. This likely reflects the increase in cases of invasive group A streptococcal infections, which may also explain the significant rise in the use of streptococcal tests and the greater prevalence of typical throat symptoms associated with this diagnosis.^27^ A decline in COVID-19 cases was noted, alongside an increase in presentations of common cold and influenza, consistent with the epidemiological trends during the respective data collection periods.^28^ This shift was accompanied by reduced use of COVID-19 rapid tests and a significant decrease in clinical findings, such as abnormal lung auscultation, tachypnoea and poor general condition. The reduction in hospital admissions and increased antibiotic prescribing for pneumonia during the second period aligns with the lower incidence of severe respiratory findings and overall clinical severity, as well as with more patients being treated at home.

The proportion of patients diagnosed with cystitis who presented with dysuria, as well as the proportion treated with first-line antibiotics, was significantly higher following the intervention. The use of narrow-spectrum first-line antibiotics for UTI was explicitly emphasized in the intervention materials, suggesting that these observed changes in UTIs may be attributable to the intervention.

Few studies have been conducted in the OOH setting, and prescription patterns often differ from those in regular-hours general practice.^29–31^ While many studies focus on prescription rates for RTIs, fewer address prescription rates per diagnosis, thereby limiting opportunities for direct comparison. Compared to a European OOH study, the prescription rate found in this study is at the higher end.^32^ Post-intervention, there appears to be a shift in aetiological labelling, with more pneumonia patients receiving antibiotics, and fewer with acute bronchitis receiving antibiotics. This may indicate increased antibiotic awareness.

The COVID-19 pandemic largely influenced the intervention, with a high proportion of antibiotic prescriptions related to COVID-19 infections before the intervention. After the intervention, this prescription rate decreased significantly. During the early stages of the COVID-19 pandemic, antibiotic prescribing was common because of limited knowledge about the virus and concerns about bacterial co-infections and may have been partly driven by patient pressure on physicians’ performance. A UK study reported a 20% prescription rate in 2020, decreasing to 12% during the third wave in 2022.^33^ In comparison, our study found a 28% prescription rate in 2022, which is relatively high given the availability of diagnostic tests and established knowledge about the virus. However, prescribing decreased significantly from 2022 to 2023. This decline may be attributed to the intervention, increased clinical experience with COVID-19, and possibly the impact of vaccination, as demonstrated in other countries.^34^

Sore throat is a common symptom for different upper respiratory infections of both viral and bacterial aetiology, and a lot of efforts have been made to guide doctors in diagnosing and prescribing only for possible bacterial infections, using CENTOR criteria and/or Strep-A test.^35–37^ Our finding of a mean Centor score <3 also among those who got a prescription indicates a substantial room for improvement of appropriate prescribing. Tonsillar exudate was a more common sign than tender cervical adenopathy. This pattern is inconsistent with the Centor criteria, which weigh all four components equally.^35^ Previous studies have likewise demonstrated that tonsillar exudate exerts a substantial influence on clinicians’ decisions to prescribe, despite the absence of symptoms or clinical signs that reliably differentiate bacterial from non-bacterial pharyngitis.^38–40^ There seems to be a widespread belief that tonsillar exudate is synonymous with bacterial infection, and this belief is likely to be reinforced during streptococcal epidemics among both physicians and patients. This important finding should be implemented in future educational programmes.

Antibiotic prescriptions were considered unnecessary when the infection was likely viral, the duration of symptoms was too short or symptom burden was minimal. There is clear potential for reduction, particularly in rhinosinusitis and otitis media, where antibiotics were prescribed after a few days of symptoms. However, the implemented intervention did not significantly influence prescribing practices for any of the infections included. The reason for this limited effect is likely multifactorial and it is unclear to what extent the COVID-19 pandemic contributed. Earlier studies have shown that many factors affect prescribing habits, including diagnostic uncertainty, pressure from patients, habits, limited time in busy OOH settings, high workload and the national and workplace culture.^3,37,38,41^ Relatively few multinational intervention studies in primary care focus on antibiotic use, and even fewer have been conducted in out-of-hours primary care settings. Researchers from both low-prescribing countries and the countries in which the study was implemented designed the present study. One potential limitation is that the intervention design may not have been fully aligned with local cultural and organizational contexts, which could partly explain why the observed effect was smaller than anticipated.

At the same time, the same intervention, implemented concurrently in general practice settings, demonstrated a greater effect.^22^ We, therefore, hypothesize that part of the explanation lies in the more heterogeneous composition of physicians working in out-of-hours care, also shown in other studies.^42,43^ This workforce includes GPs, hospital physicians and less experienced junior doctors. Furthermore, the frequent staff rotation and the limited number of permanently employed physicians make it challenging to establish consistent educational initiatives and a shared prescribing culture. Consequently, prescribing practices in out-of-hours care are likely influenced primarily by attitudes and habits acquired in physicians’ regular workplace settings.

Changing prescribing culture is also a gradual process.^44,45^ Such changes require sustained efforts over time, targeting not only physicians and healthcare professionals but also the general population. We therefore believe that studies such as ours contribute to a long-term shift in prescribing culture, although measurable effects may take time to emerge. Moreover, improvements achieved in general practice may subsequently influence prescribing behaviour in out-of-hours care, thereby facilitating gradual changes in that setting as well. Future studies should therefore continue to adopt broad, multifaceted intervention strategies while also striving to align more closely with local cultural and organizational contexts.

### Conclusions

The multifaceted intervention did not reduce overall antibiotic use for RTI but was associated with a statistically significant increase in first-line antibiotic use for cystitis. The proportion of potentially unnecessary antibiotic prescriptions remained high, indicating considerable scope for improvement. Future interventions should place greater emphasis on more clearly delineating clinical scenarios in which infections can be safely and appropriately managed without antibiotics.

## Supplementary Material

dlag219_Supplementary_Data

## Supplementary data

Figures S1 and S2 and Tables S1, S4, S7 and S8 are available as Supplementary data at *JAC-AMR* Online.

## References

[1] Goossens H, Ferech M, Vander Stichele R et al Outpatient antibiotic use in Europe and association with resistance: a cross-national database study. Lancet 2005; 365: 579–87. 10.1016/S0140-6736(05)17907-0 15708101

[2] *WHO report on surveillance of antibiotic consumption: 2016-2018 early implementation*. World Health Organization, 2018. Licence: CC BY-NC-SA 3.0 IGO.

[3] Kasse GE, Humphries J, Cosh SM et al Factors contributing to the variation in antibiotic prescribing among primary health care physicians: a systematic review. BMC Prim Care 2024; 25: 8. 10.1186/s12875-023-02223-1 PMC10759428 38166736

[4] Tonkin-Crine SKG, Tan PS, van Hecke O et al Clinician-targeted interventions to influence antibiotic prescribing behaviour for acute respiratory infections in primary care: an overview of systematic reviews. Cochrane Database Syst Rev 2017; 9: CD012252. 10.1002/14651858.CD012252.pub2 PMC6483738 28881002

[5] Cross ELA, Tolfree R, Kipping R. Systematic review of public-targeted communication interventions to improve antibiotic use. J Antimicrob Chemother 2017; 72: 975–87. 10.1093/jac/dkw520 PMC7263825 27999058

[6] Berchet C, Nader C. *The organisation of out-of-hours primary care in OECD countries. OECD Health Working Papers*. Vol. **89**. OECD Publishing, 2016.

[7] Steeman L, Uijen M, Plat E et al Out-of-hours primary care in 26 European countries: an overview of organisational models. Fam Pract 2020; 37: 744–50. 10.1093/fampra/cmaa064 PMC7699311 32597962

[8] Huibers LAMJ, Moth G, Bondevik GT et al Diagnostic scope in out-of-hours primary care services in eight European countries: an observational study. BMC Fam Pract 2011; 12: 30. 10.1186/1471-2296-12-30 PMC3114765 21569483

[9] Spurling GK, Del Mar CB, Dooley L et al Delayed antibiotic prescriptions for respiratory infections. Cochrane Database Syst Rev 2017; 9: CD004417. 10.1002/14651858.CD004417.pub5 PMC6372405 28881007

[10] European Centre for Disease Prevention and Control . Proposals for EU Guidelines on the Prudent use of Antimicrobials in Humans. ECDC, 2017. https://www.ecdc.europa.eu/sites/default/files/media/en/publications/Publications/EU-guidelines-prudent-use-antimicrobials.pdf

[11] Colliers A, Adriaenssens N, Anthierens S et al Antibiotic prescribing quality in out-of-hours primary care and critical appraisal of disease-specific quality indicators. Antibiotics 2019; 8: 79. 10.3390/antibiotics8020079 PMC6628021 31212871

[12] Colliers A, Coenen S, Bombeke K et al Understanding general practitioners’ antibiotic prescribing decisions in out-of-hours primary care: a video-elicitation interview study. Antibiotics 2020; 9: 115. 10.3390/antibiotics9030115 PMC7148451 32156082

[13] Cronberg O, Tyrstrup M, Ekblom K et al Diagnosis-linked antibiotic prescribing in Swedish primary care—a comparison between in-hours and out-of-hours. BMC Infect Dis 2020; 20: 616. 10.1186/s12879-020-05334-7 PMC7441551 32819280

[14] Lindberg BH, Gjelstad S, Foshaug M et al Antibiotic prescribing for acute respiratory tract infections in Norwegian primary care out-of-hours service. Scand J Prim Health Care 2017; 35: 178–85. 10.1080/02813432.2017.1333301 PMC5499318 28569649

[15] Lum EPM, Page K, Whitty JA et al Antibiotic prescribing in primary healthcare: dominant factors and trade-offs in decision-making. Infect Dis Health 2018; 23: 74–86. 10.1016/j.idh.2017.12.002 38715307

[16] Rose J, Crosbie M, Stewart A. A qualitative literature review exploring the drivers influencing antibiotic over-prescribing by GPs in primary care and recommendations to reduce unnecessary prescribing. Perspect Public Health 2021; 141: 19–27. 10.1177/1757913919879183 31633458

[17] Munck A, Damsgaard J, Hansen D et al The Nordic method for quality improvement in general practice. Qual Prim Care 2003; 11: 73–8. Available from: https://pure.au.dk/ portal/en/publications/the-nordic-method-for-quality-improvement-in-general-practice/.

[18] Hansen MP, Lykkegaard J, Søndergaard J et al How to improve practice by means of the Audit Project Odense method. Br J Gen Pract 2022; 72: 235–6. 10.3399/bjgp22X719417 PMC11189051 35483949

[19] Bjerrum L, Munck A, Gahrn-Hansen B et al Health alliance for prudent antibiotic prescribing in patients with respiratory tract infections (HAPPY AUDIT) -impact of a non-randomised multifaceted intervention programme. BMC Fam Pract 2011; 12: 52. 10.1186/1471-2296-12-52 PMC3146837 21689406

[20] Llor C, Cots JM, López-Valcárcel BG et al Interventions to reduce antibiotic prescription for lower respiratory tract infections: Happy Audit study. Eur Respir J 2012; 40: 436–41. 10.1183/09031936.00093211 22183489

[21] Bjerrum L, Munck A, Gahrn-Hansen B et al Health alliance for prudent prescribing, yield and use of antimicrobial drugs in the treatment of respiratory tract infections (HAPPY AUDIT). BMC Fam Pract 2010; 11: 29. 10.1186/1471-2296-11-29 PMC2877004 20416034

[22] Garcia-Sangenis A, Lykkegaard J, Hansen MP et al Impact of a multifaceted intervention programme on antibiotic prescribing and dispensing in four patient-centred settings in five European countries. The HAPPY PATIENT project. Fam Pract 2025; 42: cmae064. 10.1093/fampra/cmae064 PMC11809241 39531344

[23] Bjerrum A, Garcia-Sangenis A, Modena D et al Health alliance for prudent prescribing and yield of antibiotics in a patient-centred perspective (HAPPY PATIENT): a before-and-after intervention and implementation study protocol. BMC Prim Care 2022; 23: 102. 10.1186/s12875-022-01710-1 PMC9063370 35501712

[24] Chalkidou A, Lambert M, Cordoba G et al Misconceptions and knowledge gaps on antibiotic use and resistance in four healthcare settings and five European countries—a modified Delphi study. Antibiotics 2023; 12: 1435. 10.3390/antibiotics12091435 PMC10525245 37760731

[25] Baker R, Robertson N, Farooqi A. Audit in general practice: factors influencing participation. BMJ 1995; 311: 31–4. 10.1136/bmj.311.6996.31 PMC2550086 7613323

[26] Advani SD, Claeys K. Behavioral strategies in diagnostic stewardship. Infect Dis Clin North Am 2023; 37: 729–47. 10.1016/j.idc.2023.06.004 37537001

[27] Vieira A, Wan Y, Ryan Y et al Rapid expansion and international spread of M1UK in the post-pandemic UK upsurge of Streptococcus pyogenes. Nat Commun 2024; 15: 3916. 10.1038/s41467-024-47929-7 PMC11087535 38729927

[28] WHO COVID-19 CASES dashboard . *WHO Health Emergencies Programme*. https://data.who.int/dashboards/covid19/cases

[29] Hayward GN, Fisher RFR, Spence GT et al Increase in antibiotic prescriptions in out-of-hours primary care in contrast to in-hours primary care prescriptions: service evaluation in a population of 600 000 patients. J Antimicrob Chemother 2016; 71: 2612–9. 10.1093/jac/dkw189 PMC4992853 27287234

[30] Debets VE, Verheij TJ, van der Velden AW et al Antibiotic prescribing during office hours and out-of-hours: a comparison of quality and quantity in primary care in The Netherlands. Br J Gen Pract 2017; 67: e178–86. 10.3399/bjgp17X689641 PMC5325659 28232364

[31] Vellinga A, Luke-Currier A, Garzon-Orjuela N et al Disease-specific quality indicators for outpatient antibiotic prescribing for respiratory infections (ESAC quality indicators) applied to point prevalence audit surveys in general practices in 13 European countries. Antibiotics 2023; 12: 572. 10.3390/antibiotics12030572 PMC10044809 36978439

[32] van der Velden AW, van de Pol AC, Bongard E et al Point-of-care testing, antibiotic prescribing, and prescribing confidence for respiratory tract infections in primary care: a prospective audit in 18 European countries. BJGP Open 2022; 6: BJGPO.2021.0212. 10.3399/BJGPO.2021.0212 PMC9447323 34920989

[33] Ciaccio L, Donnan PT, Parcell BJ et al Community antibiotic prescribing in patients with COVID-19 across three pandemic waves: a population-based study in Scotland, UK. BMJ Open 2024; 14: e081930. 10.1136/bmjopen-2023-081930 PMC11033633 38643000

[34] Jorgensen SCJ, Brown K, Clarke AE et al The effect of COVID-19 vaccination on outpatient antibiotic prescribing in older adults: a self-controlled risk-interval study. Clin Infect Dis 2024; 79: 375–81. 10.1093/cid/ciae182 PMC11327793 38700036

[35] Centor RM, Witherspoon JM, Dalton HP et al The diagnosis of strep throat in adults in the emergency room. Med Decis Making 1981; 1: 239–46. 10.1177/0272989X8100100304 6763125

[36] Thai TN, Dale AP, Ebell MH. Signs and symptoms of group A versus non-group A strep throat: a meta-analysis. Fam Pract 2018; 35: 231–8. 10.1093/fampra/cmx072 29045629

[37] Cohen JF, Cohen R, Bidet P et al Efficiency of a clinical prediction model for selective rapid testing in children with pharyngitis: a prospective, multicenter study. PLoS One 2017; 12: e0172871. 10.1371/journal.pone.0172871 PMC5325561 28235012

[38] Domen J, Aabenhus R, Balan A et al The effect of a GP's perception of a patient request for antibiotics on antibiotic prescribing for respiratory tract infections: secondary analysis of a point-prevalence audit survey in 18 European countries. BJGP Open 2025; 9: BJGPO.2024.0166. 10.3399/BJGPO.2024.0166 PMC12421272 39824603

[39] Freer J, Ally T, Brugha R. Impact of centor scores on determining antibiotic prescribing in children. Int J Health Care Qual Assur 2017; 30: 319–26. 10.1108/IJHCQA-08-2016-0114 28470130

[40] Sijbom M, Buchner FL, Saadah NH et al Determinants of inappropriate antibiotic prescription in primary care in developed countries with general practitioners as gatekeepers: a systematic review and construction of a framework. BMJ Open 2023; 13: e065006. 10.1136/bmjopen-2022-065006 PMC10193070 37197815

[41] Cronberg O, Tyrstrup M, Ekblom K et al Factors influencing antibiotic prescribing for respiratory tract infections in primary care—a comparison of physicians with different antibiotic prescribing rates. Scand J Prim Health Care 2024; 42: 424–34. 10.1080/02813432.2024.2332757 PMC11332303 38625913

[42] Suttels V, Van Singer M, Clack LC et al Factors influencing the implementation of antimicrobial stewardship in primary care: a narrative review. Antibiotics 2023; 12: 30. 10.3390/antibiotics12010030 PMC9854946 36671230

[43] Zay Ya K, Win PTN, Bielicki J et al Association between antimicrobial stewardship programs and antibiotic use globally: a systematic review and meta-analysis. JAMA Netw Open 2023; 6: e2253806. 10.1001/jamanetworkopen.2022.53806 PMC9912134 36757700

[44] Soler Wenglein J, Bornemann R, Hartmann J et al Changing antibiotic prescribing cultures: a comprehensive review of social factors in outpatient antimicrobial stewardship and lessons learned from the local initiative AnTiB. Antibiotics 2025; 14: 1068. 10.3390/antibiotics14111068 PMC12649107 41301563

[45] Borek AJ, Wanat M, Atkins L et al Optimising antimicrobial stewardship interventions in English primary care: a behavioural analysis of qualitative and intervention studies. BMJ Open 2020; 10: e039284. 10.1136/bmjopen-2020-039284 PMC7747536 33334829

