# Supplementary material for: Impact of a multifaceted intervention programme on antibiotic prescribing in out-of-hours primary care in five European countries: secondary analyses of the HAPPY PATIENT project
Source: JAC Antimicrob Resist. 2026 Oct 16;8(5):dlag219. doi: 10.1093/jacamr/dlag219 (PMC13673263; doi:10.1093/jacamr/dlag219)
Supplement: Supplementary file 1 [file dlag219_supplementary_data.docx]

**Supplementary Figure S1 – Registration form**

**Supplementary Figure S2**

**Supplementary Table S1 - Description of the interventions carried out in different countries in the different settings.**

|  | Logistical details of the workshops and training sessions |
| --- | --- |
| France | Participants were dispersed geographically across France, allowing only for online interventions. A full-day online meeting (on Saturday, to allow all HCPs to participate) took place twice, with all settings at the same time, in November and December 2022. Two short online meetings were held in January 2023 for those HCPs who couldn’t attend the full-day meetings, and participants were encouraged to use the eLearning platform. |
| Greece | Online sessions, one for general practice and out-of-hours settings and another for nursing homes and pharmacy settings, were held in January 2023 for half a day. These sessions were repeated at the end of January 2023 for HCPs who could not participate in the first round, before starting the second audit. |
| Lithuania | A 2-day physical meeting was held in November 2022 (Friday afternoon and Saturday morning). HCPs from all settings were gathered together and separated into groups as needed to discuss specific topics relevant to the different settings. |
| Poland | Online sessions, one for audit and feedback results and antimicrobial resistance and one for communication training, were held in January 2023, starting in the late afternoon to allow all HCPs to participate. All settings took part in the same sessions. |
| Spain | A 1-day physical meeting was held in November 2022 for the pharmacy and general practice/nursing home settings, with groups separated by topics. Three online meetings followed in December 2022 and January 2023 for those who missed the physical meetings, divided into sessions for audit results and communication training. |

**Supplementary Table S2 - Common cold/influenza**

| Table 1 - Patients diagnosed with common cold/influenza | | | | | |
| --- | --- | --- | --- | --- | --- |
|  | **Before** | | **After** | | **P-value** |
|  | **N=347** | | **N=522** | |  |
|  | **N** | **%** | **N** | **%** |  |
| Antibiotic prescription rate* | 7 | 2.0 | 19 | 3.6 | 0.169 |
| Admission rate * | 12 | 3.5 | 16 | 3.1 | 0.748 |
| Symptom |  |  |  |  |  |
| Duration of symptoms | Mean  3.01 | SD  2.56 | Mean 2.9 | SD 2.11 | 0.394 |
| Cough | 244 | 70.3 | 387 | 74.1 | 0.216 |
| Sore throat | 204 | 58.8 | 287 | 55.0 | 0.267 |
| Purulent sputum | 16 | 4.6 | 24 | 4.6 | 0.993 |
| Fever | 165 | 47.6 | 312 | 59.8 | **<0.001** |
| Poor general condition | 34 | 9.8 | 35 | 6.7 | 0.099 |
| Clinical findings* |  |  |  |  |  |
| Abnormal lung consultation | 14 | 4.0 | 18 | 3.4 | 0.653 |
| Tachypnoea | 2 | 0.6 | 4 | 0.8 | 0.741 |
| Test performed* |  |  |  |  |  |
| CRP test performed | 103 | 29.7 | 168 | 32.2 | 0.436 |
| COVID test performed | 175 | 49.6 | 133 | 25.5 | **<0.001** |

Statistically significant: p value <0.05
*Chi-square p-Value
**T Student p-Value

**Supplementary Table S3 - COVID-19 infections**

| Table 2 - Patients diagnosed with COVID-19 | | | | | | |
| --- | --- | --- | --- | --- | --- | --- |
|  | | **Before** | | **After** | | **p-value** |
|  |  | **N=317** | | **N=111** | |  |
|  |  | N | % | N | % |  |
| Age** | | Mean  50.4 | SD 27.19 | Mean 44.3 | SD 26.57 | **0.041** |
| Antibiotic prescription rate* | | 90 | 28.4 | 6 | 5.4 | **<0.001** |
| Admission rate * | | 129 | 40.7 | 20 | 18.0 | **<0.001** |
| Double diagnose* | |  |  |  |  |  |
|  | Upper RTIs | 14 | 4.4 | 2 | 1.8 | 0.260 |
|  | Lower RTIs | 71 | 22.4 | 7 | 6.3 | **<0.001** |
| Symptom* | |  | |  | |  |
|  | Duration of symptoms** | Mean  4.6 | SD  6.26 | Mean 3.1 | SD  1.98 | **0.015** |
|  | Cough | 232 | 73.2 | 71 | 64.0 | **0.070** |
|  | Sore throat | 128 | 40.4 | 50 | 45.0 | 0.434 |
|  | Purulent sputum | 25 | 7.9 | 5 | 4.5 | 0.284 |
|  | Fever | 222 | 70.0 | 86 | 77.5 | 0.142 |
|  | Poor general condition | 103 | 32.5 | 25 | 22.5 | 0.054 |
| Clinical findings* | |  |  |  |  |  |
|  | Tachypnoea | 76 | 24.0 | 10 | 9.0 | **<0.001** |
|  | Abnormal lung consultation | 103 | 32.5 | 20 | 18.0 | **0.003** |
| Test* | |  |  |  |  |  |
|  | CRP test performed | 187 | 59.0 | 48 | 43.2 | **0.005** |
|  | COVID test performed | 291 | 91.8 | 107 | 96.4 | 0.130 |
| Patients diagnosed with COVID-19 as the only diagnose | | | | | | |
|  | | **N=229** |  | **N=102** |  | **<0.001** |
|  |  | N | % | N | % |  |
| Antibiotic prescription rate* | | 38 | 16.6 % | 2 | 2.0 % | **<0.001** |
| Admission rate * | | 70 | 30.6 % | 13 | 12.7 % | **<0.001** |
| Symptom* | |  |  |  |  |  |
| Fever | | 151 | 65.9 % | 77 | 75.5 % | 0.095 |
| Cough | | 166 | 72.5 % | 63 | 61.2 % | 0.054 |
| Purulent sputum | | 13 | 5.7 % | 4 | 3.9 % | 0.599 |
| Sore throat | | 106 | 46.3 % | 49 | 48.0 % | 0.812 |
| Poor general condition | | 65 | 28.4 % | 22 | 21.6 % | 0.224 |
| Clinical findings* | |  |  |  |  |  |
| Abnormal lung consultation | | 53 | 23.1 % | 14 | 13.7 % | 0.054 |
| Tachypnoea | | 38 | 16.6 % | 8 | 7.8 % | **0.039** |
| Test performed* | |  |  |  |  |  |
| CRP test performed | | 108 | 47.2 % | 43 | 42.2 % | 0.406 |
| COVID test performed | | 211 | 92.1 % | 98 | 96.1 % | 0.236 |

Statistically significant: p value <0.05
*Chi-square p-Value
**T Student p-Value

**Supplementary Table S4 - Acute pharyngotonsillitis**

| Table 3 – Patients diagnosed with acute pharyngotonsillitis | | | | | | |
| --- | --- | --- | --- | --- | --- | --- |
|  | | **Before** | | **After** | | **p-value** |
|  |  | N=226 | | N=302 | |  |
|  |  | N | % | N | % |  |
| Age** |  | Mean 27.9 | SD  19.04 | Mean 25.0 | SD  18.88 | 0.084 |
| Antibiotic prescription rate* | | 156 | 69.0 % | 226 | 74.8 % | 0.142 |
| Unnecessary antibiotics | | 126 | 80.8 | 201 | 88.9 | **0.025** |
| Symptom* | |  |  |  |  |  |
| Duration of symptoms** | | Mean 3.0 | SD 2.45 | Mean 3.2 | SD 2.68 | 0.553 |
| Fever | | 150 | 66.4 % | 225 | 74.5 % | 0.052 |
| Sore throat | | 204 | 90.3 % | 263 | 87.1 % | 0.274 |
| Cough | | 69 | 30.5 % | 80 | 26.5 % | 0.329 |
| Ear pain | | 20 | 8.8 % | 22 | 7.3 % | 0.520 |
| Clinical findings* | |  |  |  |  |  |
| Tonsillar exudate | | 135 | 59.7 | 189 | 62.6 % | 0.528 |
| Tender cervical adenopathy | | 72 | 31.9 | 114 | 37.7 % | 0.168 |
| Sum CENTOR Criteria’s ** | | Mean 2.27 | SD  1.05 | Mean 2.48 | SD  1.07 | **0.026** |
| CENTOR 0 | | 11 | 4.9 | 10 | 3.3 |  |
| CENTOR 1 | | 42 | 18.6 | 48 | 15.9 |  |
| CENTOR 2 | | 74 | 32.7 | 88 | 29.1 |  |
| CENTOR 3 | | 72 | 31.9 | 98 | 32.5 |  |
| CENTOR 4 | | 27 | 11.9 | 58 | 19.2 |  |
| Test performed* | |  |  |  |  |  |
| CRP test performed | | 47 | 20.8 % | 106 | 35.1 % | **<0.001** |
| Strep A test performed | | 62 | 27.4 % | 115 | 38.1 % | **0.026** |
| Type of Antibiotic prescribed* | |  |  |  |  |  |
| Penicillin or Amoxicillin in France | | 71 | 49.3 % | 107 | 52.5 % | 0.633 |
| Secondline antibiotics | | 70 | 48.6 % | 95 | 46.6 % |  |

Statistically significant: p value <0.05
*Chi-square p-Value

**T Student p-Value

**Supplementary Table S5 - Acute pneumonia**

| Table 4 – Patients diagnosed with pneumonia | | | | | |
| --- | --- | --- | --- | --- | --- |
|  | **Before** | | **After** | | **P-value** |
|  | **N=185** | | **N=163** | |  |
|  | **N** | **%** | **N** | **%** |  |
| Antibiotic prescription rate* | 147 | 79.5 | 153 | 93.9 | **<0.001** |
| Unnecessary antibiotic prescription* | 134 | 91.2 | 135 | 88.2 | 0.406 |
| Admission rate * | 133 | 71.9 | 92 | 56.4 | **0.003** |
| Duration of symptoms ** | Mean  5.83 | SD  8.01 | Mean 4.95 | SD  4.61 | 0.251 |
| Symptom* |  |  |  |  |  |
| Cough | 147 | 79.5 | 127 | 77.9 | 0.725 |
| Sore throat | 35 | 18.9 | 23 | 14.1 | 0.230 |
| Purulent sputum | 42 | 22.7 | 55 | 33.7 | **0.022** |
| Fever | 132 | 71.4 | 120 | 73.6 | 0.637 |
| Poor general condition | 92 | 49.7 | 79 | 48.5 | 0.814 |
| Clinical findings* |  |  |  |  |  |
| Abnormal lung consultation | 133 | 71.9 | 123 | 75.5 | 0.451 |
| Tachypnoea | 87 | 47.0 | 70 | 42.9 | 0.445 |
| Test performed* |  |  |  |  |  |
| CRP test performed | 152 | 82.2 | 118 | 72.4 | **0.029** |
| COVID test performed | 152 | 82.2 | 87 | 53.4 | **<0.001** |

Statistically significant: p value <0.05
*Chi-square p-Value

**T Student p-Value

**Supplementary Table S6 - Acute bronchitis/bronchiolitis**

| Table 5 Patients diagnosed with acute bronchitis | | | | | |
| --- | --- | --- | --- | --- | --- |
|  | **Before** | | **After** | | **P-value** |
|  | **N=149** | | **N=175** | |  |
|  | **N** | **%** | **N** | **%** |  |
| Antibiotic prescription rate* | 68 | 45.6 | 63 | 36.2 | 0.089 |
| Unnecessary antibiotics* | 68 | 100 | 61 | 96.8 | 0.139 |
| Admission rate * | 15 | 10.1 | 26 | 14.9 | 0.196 |
| Symptom* |  |  |  |  |  |
| Number of days with symptoms ** | Mean  5.14 | SD  4.64 | Mean 4.43 | SD  4.50 | 0.174 |
| Cough | 140 | 94.0 | 155 | 88.0 | 0.065 |
| Sore throat | 37 | 24.8 | 42 | 24.0 | 0.862 |
| Purulent sputum | 46 | 30.9 | 43 | 24.6 | 0.205 |
| Fever | 69 | 46.3 | 86 | 49.1 | 0.611 |
| Poor general condition | 14 | 9.4 | 28 | 16.0 | 0.078 |
| Clinical findings* |  |  |  |  |  |
| Abnormal lung consultation | 96 | 64.4 | 119 | 68.0 | 0.498 |
| Tachypnoea | 19 | 12.8 | 31 | 17.7 | 0.218 |
| Test performed* |  |  |  |  |  |
| CRP test performed | 67 | 45.0 | 79 | 45.1 | 0.975 |
| COVID test performed | 71 | 47.7 | 67 | 38.3 | 0.089 |

Statistically significant: p value <0.05
*Chi-square p-Value

**T Student p-Value

**Supplementary Table S7 - Acute otitis media**

| Table 6 Patients diagnosed with acute otitis media | | | | | |
| --- | --- | --- | --- | --- | --- |
|  | **N=69** |  | **N=89** |  |  |
|  | **N** | **%** | **N** | **%** |  |
| Antibiotic prescription rate* | 54 | 78.3 | 62 | 69.7 | 0.225 |
| Unnecessary antibiotics* | 48 | 88.9 | 49 | 79.0 | 0.152 |
| Admission rate * | 2 | 2.9 | 2 | 2.2 | 0.796 |
| Symptom* |  |  |  |  |  |
| Duration of symptoms** | Mean  2.7 | SD  1.95 | Mean 2.44 | SD  1.71 | 0.366 |
| Cough | 14 | 20.3 | 11 | 12.4 | 0.176 |
| Sore throat | 12 | 17.4 | 26 | 29.2 | 0.085 |
| Ear pain | 62 | 89.9 | 84 | 94.4 | 0.287 |
| Fever | 35 | 50.7 | 46 | 51.7 | 0.905 |
| Poor general condition | 3 | 4.3 | 1 | 1.1 | 0.201 |
| Clinical findings** |  |  |  |  |  |
| Abnormal lung consultation | 2 | 2.9 | 4 | 4.5 | 0.603 |
| Tachypnoea | 1 | 1.4 | 1 | 1.1 | 0.856 |
| Test performed* |  |  |  |  |  |
| CRP test performed | 19 | 27.5 | 40 | 44.9 | **0.025** |
| COVID test performed | 9 | 13.0 | 13 | 14.6 | 0.778 |

Statistically significant: p value <0.05
*Chi-square p-Value

**T Student p-Value

**Supplementary Table S8 - Acute rhinosinusitis**

| Table 7 Patients diagnosed with acute rhinosinusitis | | | | | |
| --- | --- | --- | --- | --- | --- |
|  | **Before** | | **After** | | **P-value** |
|  | **N=59** | | **N=57** | |  |
|  | **N** | **%** | **N** | **%** |  |
| Antibiotic prescription rate* | 33 | 55.9 | 36 | 63.2 | 0.455 |
| Unnecessary antibiotics* | 32 | 97.0 | 32 | 88.9 | 0.196 |
| Admission rate * | 0 | 0 | 1 | 1.8 | 0.307 |
| Symptom* |  |  |  |  |  |
| Number of days with symptoms ** | Mean 5.4 | SD  3.70 | Mean 5.9 | SD 5.37 | 0.130 |
| Cough | 30 | 50.8 | 31 | 54.4 | 0.703 |
| Sore throat | 24 | 40.7 | 29 | 50.9 | 0.270 |
| Purulent sputum | 19 | 32.2 | 15 | 26.3 | 0.486 |
| Fever | 23 | 39.0 | 26 | 45.6 | 0.470 |
| Poor general condition | 2 | 3.4 | 4 | 7.0 | 0.378 |
| Clinical findings* |  |  |  |  |  |
| Abnormal lung consultation | 5 | 8.5 | 9 | 15.8 | 0.227 |
| Tachypnoea | 0 | 0 | 0 | 0 |  |
| Test performed* |  |  |  |  |  |
| CRP test performed | 14 | 23.7 | 21 | 36.8 | 0.124 |
| COVID test performed | 26 | 44.1 | 21 | 36.8 | 0.428 |

Statistically significant: p value <0.05
*Chi-square p-Value

**T Student p-Value

**Supplementary Table S9 - Urinary tract infections**

| Table 8 Patients with only cystitis | | | | | |
| --- | --- | --- | --- | --- | --- |
|  | N=210 | | N=200 | |  |
|  | N | % | N | % | P-value |
| Sex* |  |  |  |  |  |
| Female | 178 | 84.8 % | 177 | 88.5 % | 0.267 |
| Symptom duration in days** | Mean  2.78 | SD  2.86 | Mean  2.90 | SD  4.63 | 0.765 |
| Symptom* |  |  |  |  |  |
| Dysuria | 186 | 88.6 % | 191 | 95.5 % | **<0.010** |
| Flank pain | 34 | 16.2 % | 19 | 9.5 % | **0.044** |
| Test performed* |  |  |  |  |  |
| Urinary Dip stick performed | 139 | 66.2 % | 160 | 80.0 % | **0.002** |
| CRP test performed | 47 | 22.4 % | 55 | 27.5 % | 0.231 |
| Type of antibiotic prescribed* |  |  |  |  |  |
| Narrow spectrum UTI-antibiotics | 147 | 70.0 % | 159 | 79.5 % |  |
| Broad spectrum UTI-antibiotics | 63 | 30.0 % | 41 | 20.5 % | **0.027** |
| Patients with only pyelonephritis | | | | | |
|  | N=92 | | N=90 | |  |
|  | N | % | N | % | P-value |
| Sex* |  |  |  |  |  |
| Female | 61 | 66.3 % | 56 | 62.2 % | 0.566 |
| Symptom duration in days** | Mean  3.44 | SD  3.78 | Mean  2.78 | SD  1.60 | 0.160 |
| Symptom* |  |  |  |  |  |
| Dysuria | 48 | 52.2 % | 57 | 63.3 % | 0.128 |
| Flank pain | 53 | 57.6 % | 48 | 53.3 % | 0.562 |
| Clinical finding* |  |  |  |  |  |
| Tachypnoea | 4 | 4.3 % | 5 | 5.6 % | 0.707 |
| Poor general condition | 32 | 34.8 % | 38 | 42.2 % | 0.302 |
| Test performed* |  |  |  |  |  |
| Urinary Dip stick performed | 70 | 76.1 % | 79 | 87.8 % | **0.041** |
| CRP test performed | 57 | 61.9 % | 72 | 80.0 % | **0.007** |
| Type of antibiotic prescribed* |  |  |  |  |  |
| Narrow spectrum UTI-antibiotics | 2 | 2.2 % | 7 | 7.7 % |  |
| Broad spectrum UTI-antibiotics | 90 | 97.8 % | 83 | 92.2 % | 0.081 |
| Admittance to hospital* | 35 | 38.0 % | 49 | 54.4 % | **0.026** |

Statistically significant: p value <0.05
*Chi-square p-Value

**T Student p-Value
